# Psychometric Validation of the Romanian Version of the Adult Hope Scale (AHS)

**DOI:** 10.3390/bs15070920

**Published:** 2025-07-08

**Authors:** Adriana Camelia Neagu, Sorin Ursoniu, Ion Papava, Iuliana-Anamaria Trăilă, Lavinia Palaghian, Catalina Giurgi-Oncu, Ana-Cristina Bredicean

**Affiliations:** 1 Ph.D. School, Faculty of Medicine, Department of Neuroscience, Psychiatry Discipline, “Victor Babes” University of Medicine and Pharmacy Timisoara, No. 2 E. Murgu Square, 300041 Timisoara, Romania; adriana.frantescu@umft.ro (A.C.N.); lavinia.cucer@umft.ro (L.P.); 2 “Dr. Victor Popescu” Military Emergency Clinical Hospital, 300080 Timisoara, Romania; bredicean.ana@umft.ro; 3 Department of Functional Sciences, Discipline of Public Health, Center for Translational Research and Systems Medicine, “Victor Babes” University of Medicine and Pharmacy Timisoara, No. 2 E. Murgu Square, 300041 Timisoara, Romania; sursoniu@umft.ro; 4 Department of Neuroscience, Discipline of Psychiatry, Center for Cognitive Research in Neuropsychiatric Pathology (NeuroPsy-Cog), “Victor Babes” University of Medicine and Pharmacy Timisoara, No. 2 E. Murgu Square, 300041 Timisoara, Romania; papava.ion@umft.ro (I.P.); catalina.giurgi@umft.ro (C.G.-O.); 5 Department of Pathology, ‘Pius Brînzeu’ Emergency County Clinical Hospital, 300723 Timisoara, Romania; 6 Department of Microscopic Morphology-Anatomic Pathology, ANAPATMOL Research Center, “Victor Babes” University of Medicine and Pharmacy Timisoara, 300041 Timisoara, Romania

**Keywords:** Trait Hope Scale (THS), psychometric validation, Romanian population, cultural adaptation, positive psychology

## Abstract

The present study aimed to adapt and validate the Adult Hope Scale (AHS) for the Romanian population, addressing a gap in culturally appropriate instruments for measuring dispositional hope. Based on Snyder’s theoretical model, the AHS was translated, culturally adapted, and administered to 663 Romanian adults. Psychometric evaluation included internal consistency (Cronbach’s α = 0.84 for the eight positively worded items), test–retest reliability (ICC = 0.39 for the full scale), exploratory and confirmatory factor analyses, and assessments of convergent and discriminant validity. The two-factor model of the scale (agency and pathways) was confirmed, with significant item loadings and a strong correlation between the two latent factors (r = 0.717). Convergent validity was supported by moderate correlations with the Brief Resilience Scale (r = 0.36–0.45), while discriminant validity was indicated by low correlations with the Trait Anxiety Inventory (r = 0.18–0.20). Demographic analyses revealed higher hope levels in women and engineers, with significant differences by gender (*p* = 0.00018), education (*p* = 0.031), and profession (*p* = 0.008). Despite moderate temporal stability and one weakly performing item, the Romanian AHS demonstrates strong psychometric properties, making it a valid and reliable tool for assessing hope in research and clinical settings.

## 1. Introduction

In psychology, the concept of hope has gained significant popularity, mainly due to Charles Snyder’s work, which led to the development of hope theory in the early 1990s. According to Snyder’s approach, hope is composed of three fundamental elements: agency (the drive to utilize those paths), pathways (the perceived capacity to generate numerous tactics to pursue goals), and goal setting (Snyder et al., 1991). This complex view of hope is consistent with research in other areas of psychology, suggesting that it has a connection to resilience, well-being, and particular groups going through rehabilitation or dealing with psychiatric suffering.

Although Snyder’s agency–pathways model remains the most widely used operationalization of hope, alternative conceptualizations exist. For instance, Melges’s future-oriented interventions emphasize therapeutic mechanisms focused on structuring hopes within narrative self-futuring contexts (Miao et al., 2021). Other perspectives, such as existential hope and second-wave positive psychology, consider hope within broader cultural and meaning-making frameworks (Singler, 2019; Soundy, 2025). Acknowledging these models underscores our decision to adopt Snyder’s scale due to its clear structure and extensive validation history, while situating our work within a broader theoretical framework.

Studies have supported Snyder’s hope theory, highlighting the importance of accomplishing objectives and boosting psychological resilience. Cheavens et al. demonstrated that hope is closely related to goal-oriented behaviors and the development of pathways to reach those goals (Cheavens et al., 2019). Echoing the claims of Seligman and Csikszentmihalyi regarding the promotion of hope for better psychological health, Kara and Usta also explain that hope serves as a protective factor for psychological well-being and resilience, especially during trying times like the COVID-19 pandemic (Kara & Kalay Usta, 2022). Chan et al. and Blake and Norton illustrate how hope theory is a motivational framework for enhancing life satisfaction among individuals with disabilities (Blake & Norton, 2014; Chan et al., 2013). Du outlines how nursing interventions focused on hope significantly improved the psychological states of individuals with spinal cord injuries (Du, 2024). For instance, the work of Muñoz et al. emphasizes hope’s role in coping with adverse experiences, particularly among those who have faced childhood trauma or suffer from PTSD (Munoz et al., 2020).

Trait hope refers to a stable individual characteristic involving consistent confidence in achieving goals through perceived agency (willpower) and pathways (feasible routes) (Adhikari & McLaren, 2023; Snyder et al., 2005; Wojtyna et al., 2015). In contrast, state hope describes a temporary psychological state influenced by immediate contexts and situational factors (Bernardo & Mendoza, 2021). State hope is typically assessed by evaluating an individual’s immediate perceptions of goals and agency, offering a context-sensitive measure compared to trait hope (Bernardo & Mendoza, 2021; Curry et al., 1997).

The Trait Hope Scale (THS), specifically the Adult Hope Scale (AHS), quantitatively measures trait hope using 12 items rated on an 8-point Likert-type scale. It has been translated into over 20 languages and demonstrates strong psychometric properties, including good internal consistency (α = 0.70–0.86) and test–retest reliability (Snyder et al., 2002; Vakili et al., 2022). Confirmatory factor analyses consistently validate its two-factor model of agency and pathways (Brouwer et al., 2008).

Research employing the AHS underscores its significance in various contexts, confirming the predictive value of hope for goal-directed behaviors and resilience. Clinical studies have linked higher hope to improved psychological outcomes and increased pain tolerance in rehabilitation settings (Snyder et al., 2005, 2006). It also aids adaptive coping and future orientation among patients with severe health conditions, such as stroke survivors and individuals with HIV (Gibbs et al., 2023; Oshvandi et al., 2024). In educational contexts, higher hope, as measured by the AHS, predicts academic achievement and motivation among students (Snyder et al., 2002). The scale has undergone continued validation across diverse and high-risk populations, further solidifying its status as a leading measure of dispositional hope, for example, in countries such as Spain (Galiana et al., 2015; Vinueza-Solórzano et al., 2023), France (Gana et al., 2013), Italy (Alfieri et al., 2023), China (Sun et al., 2012), Japan (Kato & Snyder, 2005), Germany (Krafft et al., 2019), Portugal (Marques et al., 2014), and Brazil (Pacico et al., 2013).

The current state of psychological testing in Romania, particularly regarding the assessment of hope, highlights a notable gap resulting from the absence of culturally validated tools. Current instruments used in Romania may fail to capture specific factors that affect hope and psychological well-being among Romanians, potentially resulting in inaccurate interpretations of mental health data (Cucu Ciuhan & Iliescu, 2022).

This study aims to psychometrically validate the Romanian adaptation of the Adult Hope Scale (AHS), providing a culturally appropriate and reliable tool for assessing trait hope among Romanian adults. Specifically, the research evaluates the translated instrument’s factorial structure, internal consistency, test–retest reliability, and convergent and discriminant validity. By filling the gap in culturally validated psychological measures, this study aims to enhance the accuracy of hope evaluation in Romania, thereby supporting more effective mental health interventions and research for the Romanian population.

## 2. Materials and Methods

### 2.1. Instruments

#### 2.1.1. Adult Hope Scale (AHS)

The Romanian version of Snyder’s Adult Hope Scale was employed, translated, and adapted specifically for this study. This scale consists of 12 items rated on a four-point Likert scale (from 1—“Almost never” to 4—“Almost always”) and is divided into two primary subscales:

- Pathways: Evaluates an individual’s ability to identify strategies for achieving their goals (items 1, 4, 6, and 8).
- Agency: Assesses motivation and determination to pursue identified goals (items 2, 9, 10, and 12).
- Four items (3, 5, 7, and 11) are filler items, reverse-scored, and, therefore, excluded from the total score calculation. The final score of the scale is computed by summing the scores of the two subscales.

#### 2.1.2. Additional Instruments for Convergent and Discriminant Validation

- Brief Resilience Scale (BRS): Comprises six items rated on a five-point Likert scale to measure an individual’s capacity to recover from stressful events.
- Trait Anxiety Inventory (STAI X2): This consists of 20 items rated on a four-point Likert scale, assessing trait anxiety as a stable personality characteristic.

### 2.2. Initial Steps

The study design was reviewed and approved by the Research Ethics Committee of the Victor Babeș University of Medicine and Pharmacy, Timișoara, Romania (Approval No. 26/03.03.2025). Before administering the assessment instruments, all participants were fully informed about the study’s nature and objectives and provided written informed consent, following applicable ethical standards and the General Data Protection Regulation (GDPR).

To initiate the validation process of the Romanian version of the Adult Hope Scale, formal written permission to use and adapt the HOPE scale was obtained from the rights holders of the late Dr. C.R. Snyder, through KU Psychology (documentation available upon request).

### 2.3. Validation Procedure

Validation of the Romanian version of the Adult Hope Scale was conducted through the following steps:

1. Content validation: The initial phase for the Romanian version of the AHS involved translating the original English version into Romanian. This translation was carried out by a psychiatrist certified in the International English Language Testing System (IELTS). A second psychiatrist, also proficient in English, performed a back-translation into English. The back-translated version was then compared with the original scale, and minor adjustments were made to ensure conceptual and linguistic equivalence. Subsequently, the third psychologists reviewed the Romanian version to assess the clarity, accuracy, and relevance of each item. Based on their feedback, problematic items were revised to enhance cultural and linguistic appropriateness. The method used was similar to that of other research groups (Roman et al., 2020; Ursoniu et al., 2021). The final Romanian version of AHS is available in Appendix A. The questionnaire was created using Google Forms (Google LLC). The survey was administered online across diverse participant groups. Participation was entirely voluntary, with no financial or material compensation offered to respondents.

2. Internal reliability and stability over time were assessed with 59 adult participants (the first session took place between 1 March 2025 and 4 March 2025), followed by a retest after ten days (the second session occurred between 14 March 2025 and 17 March 2025) to evaluate test–retest reliability and temporal stability. Two standardized instruments assessed internal reliability, including convergent and discriminant validity: BRS and the STAI-X2.

3. Main administration: AHS and additional instruments (BRS and STAI-X2) were administered to a representative sample of 663 adult participants (18 March 2025–12 May 2025). BRS and STAI-X2 tested in the main cohort will confirm AHS’s convergent and discriminant validity. The sample was diversified in age, educational level, and occupation to ensure broad representativeness.

4. Data Analysis: The collected data underwent exploratory and confirmatory factor analyses to determine factor structure. Cronbach’s alpha coefficient was calculated to determine the internal consistency of the main components of the AHS. External reliability (test–retest consistency) was evaluated using the intraclass correlation coefficient (ICC), based on repeated scale administrations over a specified time interval. Convergent and discriminant validity were assessed through Pearson correlation analyses between the scores of the AHS and those of two established instruments: the BRS and the STAI-X2. To ensure methodological rigor and to comply with best psychometric practices, the full sample of 663 participants was randomly divided into two independent subsamples. The first subsample (n = 331) was used to conduct the Exploratory Factor Analysis (EFA), while the second subsample (n = 332) was reserved for the Confirmatory Factor Analysis (CFA). This division prevented overlap between the exploratory and confirmatory phases, thereby enhancing the robustness of the factor structure validation.

### 2.4. Population and Questionnaire Administration

To validate the AHS, three datasets were collected: a pilot test dataset (n_1_ = 59 participants), a retest dataset applied to the same sample as the pilot dataset (n_2_ = 59 participants), and a third validation dataset collected from n_3_ = 663 participants.

Individuals from diverse social backgrounds, of various ages, and from different regions of the country participated in the study. Data collection occurred between 20 February 2025, and 12 May 2025. The first section of the questionnaire included instructions for completion, a description of the study’s purpose, and the informed consent statement. The following section gathered demographic information. Section 2 contained the AHS, while Section 3 and Section 4 included the BRS and the STAI-X2. The questionnaire was self-administered online using the Google Forms platform (https://docs.google.com/forms/d/1wmnLGB2bkbiidm67tBT7hVcc5qDKpr_TFj1-NAnX4VU/edit (accessed on 3 June 2025)). To ensure participant anonymity, each respondent was asked to enter a unique code composed of the initials of their name and the last three digits of their personal identification number.

### 2.5. Statistical Software Tools Used

#### 2.5.1. Statistical Analyses Were Conducted Using the Following:

- JASP (version 0.18.3): An open-source software for performing exploratory and confirmatory factor analyses;
- Python (version 3.12): Utilized for additional statistical analysis, data processing, and visualization, leveraging statistical and graphical libraries (e.g., pandas, NumPy, matplotlib, and scikit-learn).

A Cronbach’s alpha coefficient of ≥0.7 indicated acceptable internal consistency. Test–retest reliability was assessed using the ICC, with values ≥0.3 considered acceptable. *p*-value < 0.05 was considered statistically significant.

#### 2.5.2. Disclaimer Regarding the Use of AI Tools and Digital Platforms

The preparation and editing of this article involved the following artificial intelligence tools and digital platforms:

- ChatGPT (GPT-4, March 2024 version): Used for linguistic editing, proofreading, and structural enhancement of the text.
- Consensus.app (latest available version as of March 2024) and Scite.ai (latest available version as of March 2024): Utilized for literature review, identification of scientific consensus, and validation of cited references.

## 3. Results

### 3.1. Internal Consistency, Temporal Stability, and Convergent and Discriminant Validity

AHS exhibits variable psychometric properties across its item sets (Table 1). For the full 12-item scale, internal consistency, measured by Cronbach’s alpha (α = 0.65), indicates moderate reliability. This suggests that including positively and negatively worded items may have introduced some internal inconsistency. In contrast, the subscale of positively worded items shows excellent internal consistency (α = 0.85), supporting the reliability of these items in assessing the construct of hope. The negatively worded items yield a lower but acceptable Cronbach’s alpha (α = 0.68), suggesting modest internal reliability.

As assessed by the ICC, test–retest reliability reveals low to moderate consistency over time. The ICC values are 0.39 for the full scale, 0.30 for the positive items, and 0.34 for the negative items. While all values exceed the minimal acceptability threshold (ICC ≥ 0.30), they indicate only fair stability.

Empirical evidence supports the construct validity of the Romanian version of AHS through convergent and discriminant validation procedures (Table 2). The Pearson correlation coefficients for the test dataset show a moderate positive correlation (r = 0.42) between AHS scores and scores from the BRS, supporting convergent validity. This correlation increases slightly in the retest dataset (r = 0.45).

Contrary to theoretical expectations, the correlation between the Adult Hope Scale and the Trait Anxiety Inventory (STAI-X2) was found to be low and positive. Although prior research has consistently reported a negative association between hope and anxiety, our findings may reflect measurement overlap, reversed item effects, or cultural differences in the interpretation of dispositional hope and anxiety.

### 3.2. Demographic Factors Vs. Scores of AHS

The assessment of AHS performance was carried out on a larger and more diverse sample, the main lot, n_3_ = 663 participants.

Table 3 represents AHS scores by demographic factors. Female participants reported significantly higher levels of hope than males, with a statistically significant difference (p = 0.00018), indicating gender-based variation in trait hope. There were no statistically significant differences in AHS scores across age groups (*p* = 0.944). Median scores were consistent across the 18–30, 31–60, and 60+ age brackets, suggesting age does not influence hope levels in this sample.

Educational background had a significant impact on hope levels (*p* = 0.031). Individuals with only a high school education exhibited the highest median AHS score, followed by those with secondary education (and university graduates, indicating a slight inverse relationship between education level and hope. Significant differences were found across professions (*p* = 0.008). Engineers displayed the highest levels of hope, while psychologists reported the lowest. These findings suggest that occupational context may meaningfully shape individuals’ dispositional hope.

### 3.3. Convergent and Discriminant Validity in the Main Lot

The study aimed to reconfirm the convergent and discriminant validity of the AHS in the larger sample (Table 4); therefore, the BRS and STAI-X2 were also administered to this group. The Pearson correlation coefficient for convergent validity is 0.36, indicating a moderate positive relationship between the AHS and the BRS. The discriminant validity is confirmed by a low correlation (r = 0.18) between the AHS and the STAI-X2, which indicates that the AHS assesses a construct distinct from trait anxiety.

### 3.4. Assessment of the AHS’s Performance

The Cronbach’s Alpha internal consistency coefficient calculated for the AHS (eight scalable items), excluding the four filler items, was α = 0.84. This value indicates good internal reliability for solid psychometric use.

To confirm the suitability of the data for factor analysis, Kaiser–Meyer–Olkin (KMO) measure of sampling adequacy (KMO = 0.83) and Bartlett’s test of sphericity (χ^2^(28) = 524.17, *p* < 0.001) were used, indicating sufficient intercorrelations among items. EFA was conducted using the Maximum Likelihood extraction method with Varimax rotation. The number of retained factors was determined using multiple criteria: eigenvalues greater than 1 (Kaiser’s criterion), inspection of the scree plot, and parallel analysis. All supported a two-factor solution consistent with Snyder’s theoretical model. All items showed strong primary loadings (≥0.40) and minimal cross-loadings (<0.30), except item X, which will be considered for refinement in future studies. Communalities ranged from 0.52 to 0.79, indicating substantial shared variance.

The EFA results indicate a two-factor structure consistent with Snyder’s original theoretical model, identifying distinct dimensions of Agency (Factor 1) and Pathways (Factor 2) (Table 5). Items such as item 1 (loading = 0.74) and item 2 (loading = 0.69) loaded strongly on the Agency factor, reflecting motivational aspects of hope. Conversely, item 4 (loading = 0.68) and item 6 (loading = 0.71) loaded on the Pathways factor, indicating cognitive–strategic components. Some cross-loadings and lower secondary loadings (e.g., item 12 loading = 0.24 on Pathways) suggest possible conceptual overlaps or interpretation variability in the Romanian adaptation. Overall, the structure supports the theoretical two-factor model of the scale.

Confirmatory Factor Analysis (CFA) was conducted using the Maximum Likelihood Estimation (MLE) method (Table 6). The assumption of multivariate normality was assessed and found to be acceptable. The initially tested two-factor correlated model showed good fit to the data: χ^2^(19) = 34.21, *p* = 0.021, CFI = 0.970, TLI = 0.951, RMSEA = 0.045 [90% CI: 0.020–0.068], and SRMR = 0.035. All fit indices met conventional benchmarks for acceptable model fit (CFI/TLI ≥ 0.90, RMSEA and SRMR ≤ 0.08). Given the non-significant loading of item 6, a second model was tested excluding this item. The revised model demonstrated improved fit: χ^2^(13) = 20.89, *p* = 0.077, CFI = 0.985, TLI = 0.971, RMSEA = 0.037 [90% CI: 0.000–0.067], and SRMR = 0.029. This suggests that removing item 6 may enhance the structural validity of the instrument.

The CFA results confirm the two-factor model of the Romanian AHS, with significant standardized estimates for most items loading on their respective latent factors—Agency and Pathways. All factor loadings are statistically significant (*p* < 0.001), except for item 6 (Estimate = −0.022, *p* = 0.862), indicating this item does not significantly contribute to the Pathways factor and may require revision or removal. Item loadings for Agency range from 0.375 to 0.977, while for Pathways they range from −0.79 to 1.147, indicating moderate to strong relationships, with the exception noted. The two latent factors, Agency and Pathways, are positively correlated (Estimate = 0.717, *p* < 0.001), supporting the theoretical interdependence within the hope construct. The model also shows strong reliability of the latent constructs, with variances of 0.995 (Agency) and 0.668 (Pathways), respectively. Overall, the CFA provides robust statistical support for the Romanian AHS’s construct validity, highlighting one problematic item (item 6) that may undermine measurement accuracy.

## 4. Discussion

Hope is a central psychological construct associated with adaptive functioning, resilience, and well-being across diverse populations. Globally, hope has been linked to adaptive functioning, resilience in the face of adversity, and improved psychological well-being (Duggal et al., 2016; Lima et al., 2023; Velez et al., 2024; Yıldırım & Arslan, 2022). Defined by Snyder as a future-oriented mindset involving both agency (goal-directed energy) and pathways (planning to meet goals), hope has demonstrated relevance in various domains, including mental health, education, and occupational settings (Feldman & Jazaieri, 2024; Murphy, 2023; Snyder et al., 1996; Velez et al., 2024; Wong & Cheung, 2024).

This study is the first to adapt and validate the AHS for Romanian adults. Our findings confirm that the Romanian version of the AHS maintains good psychometric properties, especially for the positively worded subscale. The full scale’s Cronbach’s alpha of 0.84 aligns well with international benchmarks, supporting internal consistency. However, internal reliability for the negatively worded items was modest (α = 0.68), reflecting a recurring issue in cross-cultural adaptations that include reverse-scored items. As measured by Cronbach’s alpha, internal consistency has been reported as satisfactory in previous validations conducted across international contexts, with a value of 0.91 for the USA population (Snyder et al., 1996). This suggests that future revisions might benefit from either rephrasing or removing such items to minimize response bias and enhance interpretability.

Regarding temporal stability, assessed through intraclass correlation coefficients (ICCs), it was lower than expected. The total score and subscales demonstrated limited consistency over a 9-day interval. This fluctuation may reflect either situational influences or difficulties in processing reverse-coded items—an issue documented in other cross-cultural validations (Gallagher & Lopez, 2009). This finding raises questions about the suitability of AHS for longitudinal use in its current form.

Construct validity was assessed through convergent and discriminant correlations with validated external instruments. The moderate positive correlation between the AHS and the BRS supports convergent validity, indicating that dispositional hope aligns meaningfully with psychological resilience. However, the correlation between the AHS and the STAI-X2 was low and unexpectedly positive across both test and retest conditions (Arnau et al., 2007; Gallagher & Lopez, 2009). While this result may initially appear to support discriminant validity, it diverges from prior literature that typically reports a negative relationship between hope and anxiety. This discrepancy warrants cautious interpretation. One possible explanation lies in conceptual or semantic overlap between hope and introspective concern in the Romanian cultural context, or in the potential impact of reverse-coded items in the STAI-X2, which may have influenced participant responses. Future studies should investigate this association further, examining cultural and linguistic factors that may shape how affective traits are self-reported and understood.

Demographic analyses revealed that women reported higher levels of hope than men, consistent with prior research suggesting gender differences in emotional awareness and future orientation (Bazargan-Hejazi et al., 2023; Casile et al., 2021; Gupta et al., 2019; Moudatsou et al., 2025). Age, on the other hand, did not significantly influence hope scores, supporting the idea that dispositional hope is a stable trait throughout the adult lifespan, as demonstrated in other studies (Dervis et al., 2022; Long et al., 2020).

A surprising trend emerged regarding education: participants with lower formal education reported higher levels of hope. This finding may reflect sociocultural dynamics in Romania, where unmet expectations in higher-educated groups, particularly in a volatile labor market, could reduce dispositional optimism (Chipperfield et al., 2019; Dervis et al., 2022). Occupational differences further underscored this complexity—engineers reported the highest hope levels, potentially due to the problem-solving orientation and stability of their profession. At the same time, psychologists scored the lowest, perhaps indicating emotional strain or burnout associated with their field (Hussien et al., 2021; Schubert et al., 2023).

The results of the EFA and CFA provide robust support for the two-factor model of the Romanian version of the Adult Hope Scale (AHS), in line with Snyder’s original theoretical framework, which conceptualizes hope as comprising two interrelated components: agency and pathways. The EFA, conducted on half of the sample (n = 331), showed that positively worded items loaded clearly onto their intended factors. For instance, item 1 loaded strongly on the Agency factor (loading = 0.74), and item 6 showed a strong association with the Pathways factor (loading = 0.71). These findings confirm the structural validity of the two-dimensional model. However, minor cross-loadings and lower secondary loadings suggest possible cultural or semantic nuances in the Romanian adaptation that warrant further examination.

CFA conducted on the second half of the sample (n = 332) confirmed the two-factor correlated model using MLE. Most items had statistically significant standardized loadings, except for item 6, which did not load significantly on the Pathways factor, indicating problematic functioning and recommending reconsideration for future use. Fit indices demonstrated excellent model fit. When item 6 was excluded, model fit improved further, indicating its exclusion may enhance construct validity. The two latent constructs—Agency and Pathways—were positively correlated, supporting their interdependence within Snyder’s hope theory. These results affirm the theoretical coherence and psychometric integrity of the Romanian AHS, with one item (item 6) flagged for refinement.

These factor-analytic findings confirm that the Romanian AHS preserves the conceptual fidelity of Snyder’s hope theory while highlighting one item (item 6) that warrants psychometric adjustment. This result suggests that the item does not align well with the latent construct it is intended to measure, thereby weakening the overall construct validity of the Romanian AHS. Its inclusion could introduce measurement noise, reduce model fit, and obscure true relationships with external variables. One possible explanation may lie in semantic ambiguities or cultural differences in how goal-planning is conceptualized in Romanian society. Alternatively, the item’s phrasing may lack clarity or emotional resonance with respondents. Given these concerns, we strongly recommend further investigation using both psychometric refinements and qualitative methods (e.g., cognitive debriefing) to determine whether rewording or exclusion is more appropriate.

This study is not without limitations. First, although the sample size was substantial and demographically diverse, the data collection method relied exclusively on online self-report, which may introduce response biases and limit participation to individuals with access to and literacy in digital technology. Second, while the Romanian version of the AHS demonstrated strong internal consistency for the positively worded items, reverse-scored items negatively impacted overall reliability and interpretability, suggesting potential cultural or linguistic challenges in item formulation. The third limitation of this study is the absence of outcome variables that would allow for a formal test of predictive validity. Additionally, the low temporal stability observed across test–retest intervals highlights the influence of situational variability, which may limit the scale’s robustness for longitudinal applications.

Future research should refine or eliminate psychometrically weak items, such as item 6, and validate the scale in clinical or at-risk groups. Exploring the predictive role of hope in resilience, coping, and mental health through longitudinal designs would strengthen the scale’s practical utility. Qualitative methods could further improve cultural adaptation, ensuring clarity and relevance in Romanian populations.

## 5. Conclusions

This study provides the first psychometric validation of the AHS for use in the Romanian population, offering a culturally adapted and theoretically robust tool for measuring dispositional hope. The findings confirm the scale’s two-factor model and demonstrate strong internal consistency, particularly for positively worded items. Despite moderate temporal stability, the scale shows solid construct validity, with meaningful correlations to resilience and low association with trait anxiety. Demographic analyses revealed significant differences in hope levels based on gender, education, and profession, offering culturally relevant insights into how hope manifests in Romanian society. While one item (item 6) requires refinement due to weak factor loading, the overall results support the AHS as a reliable and valid instrument. This validation fills a gap in Romanian psychological assessment, enabling future research and clinical work to more accurately evaluate and support the construct of hope in both general and applied settings.

## Figures and Tables

**Table 1:** Internal consistency and temporal stability (n_1_ = 59, n_2_ = 59).

	No. of Items	Cronbach’s Alpha	ICC (Test–Retest)
All AHS items	12	0.65	0.39
Positive items	8	0.85	0.3
Negative items	4	0.68	0.34

**Table 2:** Convergent and discriminant validity (n_1_ = 59, n_2_ = 59).

	Test Dataset (Pearson r)	Retest Dataset (Pearson r)
Convergent	0.42	0.45
Discriminant	0.2	0.2

**Table 3:** AHS scores per demographic factors (n_3_ = 663).

Demographic Factors		Overall Median and (Interquartile Range—IR)
AHS total score		19 (17–22)
Gender	Male	18 (16–21)
	Female	20 (17–23)
	*p*-value *	0.00018
Age category	18–30 years	19 (16–22)
	31–60 years	19 (17–22)
	60+ years	19 (18–23)
	*p*-value **	0.944
Education	High school	20.5 (17–23)
	Secondary education	20 (17.25–22)
	University education	19 (16–22)
	*p*-value **	0.031
Profession	Healthcare provider	20 (16–22)
	Psychologist	17 (15–19)
	Economist	18 (17–21)
	Engineer	24 (18–25.5)
	Teacher	20 (17–23)
	Student	20 (18–23)
	Other	19 (17–22)
	*p*-value **	0.008

* Mann–Whitney U Test. ** Kruskal–Wallis Test.

**Table 4:** Convergent and discriminant validity in the main lot (n_3_ = 663).

	Main Dataset (Pearson r)
Convergent	0.36
Discriminant	0.18

**Table 5:** Exploratory Factor Analysis (EFA) for the Romanian version of AHS (eight positive items), the EFA subsample (n_3_ = 330).

Item (Short)	Factor 1—Agency	Factor 2—Pathways
I can think of many ways to get out (item 1)	0.74	0.21
I energetically pursue my goals (item 2)	0.69	0.25
There are lots of ways around any problem (item 4)	0.23	0.68
I can think of many ways to get the things in life that are important (item 6)	0.27	0.71
I know I can find a way to solve the problem (item 8)	0.33	0.65
My past experiences have prepared me well for my future (item 9)	0.61	0.29
I’ve been pretty successful in life (item 10)	0.66	0.19
I meet the goals that I set for myself (item 12)	0.70	0.24

**Table 6:** Confirmatory Factor Analysis (CFA) for the Romanian version of AHS (8 positive items), the CFA Subsample (n_3_ = 333).

LHS	OP	RHS	Estimate	Std. Err	z-Value	*p*-Value
item2	~	Agency	1.000	-	-	-
item9	~	Agency	−0.551	0.1027	−5.362	<0.001
item10	~	Agency	0.977	0.1027	14.046	<0.001
item12	~	Agency	0.375	0.0487	7.701	<0.001
item1	~	Pathways	1.000	-	-	-
item4	~	Pathways	−0.79	0.1277	−6.185	<0.001
item6	~	Pathways	−0.022	0.1243	−0.1744	0.862
item8	~	Pathways	1.147	0.1230	9.327	<0.001
Agency	~~	Agency	0.995	0.1052	9.4589	<0.001
Pathways	~~	Pathways	0.668	0.1183	5.642	<0.001
Pathways	~~	Agency	0.717	0.0814	8.816	<0.001
item1	~~	item1	1.942	0.1274	15.245	<0.001
item10	~~	item10	0.765	0.0708	10.810	<0.001
item12	~~	item12	1.022	0.0586	17.436	<0.001
item2	~~	item2	0.753	0.0724	10.396	<0.001
item4	~~	item4	3.612	0.2092	17.263	<0.001
item6	~~	item6	4.810	0.2648	18.165	<0.001
item8	~~	item8	1.393	0.1150	12.106	<0.001
item9	~~	item9	4.975	0.2788	17.844	<0.001

LHS—Left-Hand Side (The variable being predicted or explained). OP—Operator (Indicates the type of relationship: ~: regression/factor loading, ~~: variance/covariance). RHS—Right-Hand Side (The predictor variable).

## Data Availability

The data supporting this study are available from the corresponding author upon request. However, due to ethical considerations, they are not publicly accessible.

## Abbreviations

The following abbreviations are used in this manuscript:

AHS	Adult Hope Scale
THS	Trait Hope Scale
BRS	Brief Resilience Scale
CFI	Comparative Fit Index
df	Degrees of freedom
STAI-X2	Trait Anxiety Inventory–Form X2
ICC	Intraclass Correlation Coefficient
MLE	Maximum Likelihood Estimation
EFA	Exploratory Factor Analysis
CFA	Confirmatory Factor Analysis
RMSEA	Root Mean Square Error of Approximation
SRMR	Standardized Root Mean Square Residual
GDPR	General Data Protection Regulation
TLI	Tucker–Lewis Index

## Appendix A

### Romanian Version of the Trait Hope Scale

Scala Sperantei ca trasatura Synder

1. Mă pot gândi la multe moduri de a scăpa dintr-o încurcătură.

2. Îmi urmăresc cu determinare îndeplinirea obiectivelor.

3. Mă simt obosit, în cea mai mare parte a timpului.

4. Există mai multe moduri pentru a rezolva o problemă.

5. Pierd ușor într-o dispută.

6. Mă pot gândi la multe modalități de a obține lucrurile importante pentru mine.

7. Îmi fac griji pentru sănătatea mea.

8. Chiar și atunci când ceilalți sunt descurajați, știu că pot găsi o modalitate de a rezolva problema.

9. Experiențele mele din trecut m-au pregătit bine pentru viitor.

10. Am avut destul de mult succes în viață.

11. De obicei, îmi fac griji pentru orice.

12. Îmi îndeplinesc obiectivele pe care mi le-am propus.

Ce măsoară chestionarul?

Scala speranței pentru adulți (AHS) măsoară modelul cognitiv al speranței propus de Snyder, care definește speranța ca fiind “un stadiu motivațional pozitiv bazat pe un sentiment interactiv de reușită în ceea ce privește (a) agenția (energie orientată spre obiective) și (b) căile (planificarea pentru atingerea obiectivelor)”.

Scala speranței pentru adulți conține 12 itemi. Patru itemi măsoară gândirea legată de căi, patru itemi măsoară gândirea legată de agenție, iar patru itemi sunt de umplutură. Participanții răspund la fiecare item folosind o scală de 8 puncte, variind de la “complet fals” la “complet adevărat”, iar completarea scalei durează doar câteva minute. Pentru o analiză detaliată a teoriei speranței și a cercetărilor aferente, vezi Snyder et al (2002).

Structura Scalei Speranței pentru Adulți:

Subscala “Pathways” (Căi): Evaluează capacitatea individului de a genera rute către atingerea obiectivelor.

Itemii: 1, 4, 6, 8

Subscala “Agency” (Agenție): Evaluează motivația și determinarea de a urma aceste rute.

Itemii: 2, 9, 10, 12

Itemi de umplutură: Nu sunt utilizați în calculul scorului total.

Itemii: 3, 5, 7, 11

Itemii retroversi (formulați negativ) din Scala Speranței sunt:

- 3. Mă simt obosit, în cea mai mare parte a timpului.
- 5. Pierd ușor într-o dispută.
- 7. Îmi fac griji pentru sănătatea mea.
- 11. De obicei, îmi fac griji pentru orice.

Acești itemi sunt formulați într-un mod care reflectă pesimismul, îngrijorarea sau lipsa de speranță.

Instrucțiuni pentru completare:

Participanții sunt rugați să citească fiecare afirmație și să indice, pe o scală de la 1 la 8, cât de adevărată este acea afirmație pentru ei, unde 1 înseamnă „Complet fals” și 8 înseamnă „Complet adevărat”.

Calcularea scorului:

Subscala „Pathways”: Se adună scorurile itemilor 1, 4, 6 și 8.

Subscala „Agency”: Se adună scorurile itemilor 2, 9, 10 și 12.

Scorul total al speranței: Se adună scorurile celor două subscale.

## Appendix B

### English Version of the Trait Hope Scale

The Scale of Hope as a Trait of Synder

1. I can think of many ways to get out of a jam.

2. I energetically pursue my goals.

3. I feel tired most of the time.

4. There are lots of ways around any problem.

5. I am easily downed in an argument.

6. I can think of many ways to get the things in life that are important to me.

7. I worry about my health.

8. Even when others get discouraged, I know I can find a way to solve the problem.

9. My past experiences have prepared me well for my future.

10. I’ve been pretty successful in life.

11. I usually find myself worrying about something.

12. I meet the goals that I set for myself.

What does the questionnaire measure?

The Adult Hope Scale (AHS) measures the cognitive model of hope proposed by Snyder, who defines hope as “a positive motivational state based on an interactively derived sense of successful (a) agency (goal-directed energy) and (b) pathways (planning to meet goals).”

The Adult Hope Scale consists of 12 items. Four items measure pathway thinking, four items measure agency thinking, and four items are fillers. Participants respond to each item using an 8-point scale ranging from “definitely false” to “definitely true,” and completing the scale takes only a few minutes. For a detailed analysis of hope theory and related research, see Snyder et al (2002).

Structure of the Adult Hope Scale:

“Pathways” Subscale: Assesses an individual’s ability to generate routes toward achieving goals.

Items: 1, 4, 6, 8

“Agency” Subscale: Assesses the motivation and determination to pursue those routes.

Items: 2, 9, 10, 12

Filler Items: Not used in calculating the total score.

Items: 3, 5, 7, 11

Negatively worded (reverse-scored) items in the Hope Scale are:

- 3. I feel tired most of the time.
- 5. I am easily downed in an argument.
- 7. I worry about my health.
- 11. I usually find myself worrying about something.

These items are phrased in a way that reflects pessimism, worry, or lack of hope.

Instructions for Completion:

Participants are asked to read each statement and indicate, on a scale from 1 to 8, how true that statement is for them, where 1 means “Definitely False” and eight means “Definitely True.”

Scoring:

Pathways Subscale: Add the scores of items 1, 4, 6, and 8.

Agency Subscale: Add the scores of items 2, 9, 10, and 12.

Total Hope Score: Sum the scores of both subscales.
